# Characterizing the Hot Spots Involved in RON-MSPβ Complex Formation Using *In Silico* Alanine Scanning Mutagenesis and Molecular Dynamics Simulation

**DOI:** 10.15171/apb.2017.018

**Published:** 2017-04-13

**Authors:** Omid Zarei, Maryam Hamzeh-Mivehroud, Silvia Benvenuti, Fulya Ustun-Alkan, Siavoush Dastmalchi

**Affiliations:** ^1^Department of Pharmaceutical Biotechnology, Faculty of Pharmacy, Tabriz University of Medical Sciences, Tabriz, Iran.; ^2^Biotechnology Research Center, Tabriz University of Medical Sciences, Tabriz, Iran.; ^3^Students Research Committee, Tabriz University of Medical Sciences, Tabriz, Iran.; ^4^Department of Medicinal Chemistry, Faculty of Pharmacy, Tabriz University of Medical Sciences, Tabriz, Iran.; ^5^Molecular Therapeutics and Exploratory Research Laboratory, Candiolo Cancer Institute-FPO-IRCCS, Candiolo, Turin, Italy.; ^6^Department of Pharmacology and Toxicology, Faculty of Veterinary Medicine, Istanbul University, Istanbul, Turkey.

**Keywords:** Alanine screening, Cancer, Drug design, Molecular dynamic simulation, MSP, RON

## Abstract

***Purpose:*** Implication of protein-protein interactions (PPIs) in development of many diseases such as cancer makes them attractive for therapeutic intervention and rational drug design. RON (Recepteur d’Origine Nantais) tyrosine kinase receptor has gained considerable attention as promising target in cancer therapy. The activation of RON via its ligand, macrophage stimulation protein (MSP) is the most common mechanism of activation for this receptor. The aim of the current study was to perform in silico alanine scanning mutagenesis and to calculate binding energy for prediction of hot spots in protein-protein interface between RON and MSPβ chain (MSPβ).

***Methods:*** In this work the residues at the interface of RON-MSPβ complex were mutated to alanine and then molecular dynamics simulation was used to calculate binding free energy.

***Results:*** The results revealed that Gln^193^, Arg^220^, Glu^287^, Pro^288^, Glu^289^, and His^424^ residues from RON and Arg^521^, His^528^, Ser^565^, Glu^658^, and Arg^683^ from MSPβ may play important roles in protein-protein interaction between RON and MSP.

***Conclusion:*** Identification of these RON hot spots is important in designing anti-RON drugs when the aim is to disrupt RON-MSP interaction. In the same way, the acquired information regarding the critical amino acids of MSPβ can be used in the process of rational drug design for developing MSP antagonizing agents, the development of novel MSP mimicking peptides where inhibition of RON activation is required, and the design of experimental site directed mutagenesis studies.

## Introduction

Protein-protein interactions (PPIs) are involved in many biological processes as key regulatory steps^1^ and when aberrantly regulated are implicated in the development of many diseases such as cancer.^2-4^ These versatile roles make PPIs attractive for therapeutic intervention and rational drug design.^5-7^ Different classes of the approved therapeutic agents or those in development stages have been shown to interfere with PPIs in order to overcome the corresponding diseases.^8-11^

In PPI, the amino acids at the interaction interface have great importance in terms of starting point for the initiation of the biological and cellular functions.^12^ Usually several residues are exposed at the interface between the interacting proteins, but they do not contribute equally to the binding energy. The important key residues when mutated into alanine residue weaken the binding strength (increase of free energy of binding at least 2.0 kcal/mol) are called “hot spots”.^13,14^ Identification of the hot spots is critical in designing therapeutic agents which exert their effects by influencing PPIs. One of the experimental methods commonly used for identification of these hot spots is site-directed mutagenesis followed by comparative functional assays of the mutated and the wild type proteins; however, these experiments are time-consuming and expensive.^15^ Structural elucidation of the partner proteins within a complex by means of biophysical methods such as X-ray crystallography and NMR is also possible but again costly and demanding.^16^ In the era of modern drug discovery and development, the use of *in silico* methods shortens the rational drug design process in terms of both time and cost.^17-21^ In this regard, identifying hot spots is not an exception.^22,23^ Computational alanine scanning mutagenesis is a virtual method which has been extensively used for the characterization and prediction of hot spots in protein-protein, protein-DNA and protein-small molecule complexes.^22,24-29^ Charged, polar, or bulky amino acids are virtually mutated to a neutral, small and non-polar amino acid such as alanine and then binding free energy is calculated for both wild type and mutant forms in order to estimate the contribution of the mutated residues to the binding energy.^30,31^ One of the most routinely used approaches for computational estimation of binding free energy is based on accessible surface area models of implicit solvation method where molecular mechanics data are treated by Generalized Born surface area (MM-GBSA) algorithm.^32-40^

Tyrosine kinase receptors (TKRs) involved in well characterized protein-protein interactions are among potential candidate targets for anticancer drug development.^41-46^ TKRs are cell surface receptors for different polypeptide ligands and have pivotal roles in regulation of many cellular functions and physiological events^47,48^ and when aberrantly expressed and activated play key functions in development and progression of different types of cancers.^49-54^ Ligand-mediated receptor dimerization is the main mechanism of activation triggered by ligand binding to the extracellular domain of its specific receptor.^55-57^ This protein-protein interaction causes receptor dimerization followed by authophosphorylation of tyrosine residues located within the intracellular tyrosine kinase domain (catalytic tyrosines) followed by phosphorylation of tyrosine residues located within the C tail (docking tyrosines) that become the docking site for adaptor/effector proteins responsible of transducing the downstream signaling pathways resulting in cellular proliferation, differentiation, metabolism, survival, migration, and cell cycle control.^58^ In principle, all PPIs mediated by TKRs (including the downstream PPIs) could be targeted for cancer therapy^59,60^ but generally therapeutic PPI inhibitors interfere with the binding of endogenous ligands to the receptor.^61-67^ Therefore, it is obvious that uncovering the details of PPIs between TKRs and their ligands can provide useful information applicable to design of new anticancer agents.

RON (Recepteur d'Origine Nantais) is a member of TKRs superfamily, its role in tumorigenesis has been established in different cancer types and numerous studies have suggested RON as a promising target for anticancer drug development.^68,69^ RON also known as MSTR1 (Macrophage Stimulating Receptor1) belongs to MET proto-oncogene family,^70^ and is usually expressed at low levels in normal tissues while it is highly expressed in cancer cells.^71^ Structurally, RON is a disulfide linked heterodimer protein made of two chains, an extracellular α-chain and a β-chain which consists extracellular, transmembrane, and intracellular regions. The extracellular domain comprises three distinct domains including Sema, Plexin-Semaphorin-Integrin (PSI), and three Immunoglobulin-Plexin-Transcription factor (IPT1-IPT3) domains.^68^ The natural ligand of RON is MSP (Macrophage Stimulating Protein),^72^ a member of plasminogen-related kringle protein family^73^ which is a heterodimeric protein made of an α-chain composed of four kringle domains and a β-chain containing a serine protease-like domain.^74^ The α- and β-chains of MSP show low and high affinities to RON Sema domain, respectively.^75^ Several monoclonal antibodies against RON extracellular domain have been developed (in preclinical phases) to specifically inhibit the protein-protein interactions between RON and MSP.^69^ Identifying the key residues working as hot spots responsible for receptor-ligand (RON-MSP) interaction is of great importance for drug design and development. The aim of the current study is to identify hot spots involved in RON-MSPβ interaction using *in silico* alanine scanning mutagenesis by MM-GBSA method. The results can be used in anticancer drug designing where inhibition of RON is needed.

## Materials and Methods

### Structure preparation and in silico alanine mutagenesis

Experimental coordinates of RON complexed with MSPb (PDB ID: 4QT8) determined at 3.0 Å resolution by X-ray crystallography^76^ was retrieved from the Protein Data Bank at the Research Collaboratory for Structural Bioinformatics (http://www.rcsb.org/pdb/ home/home.do).^77^ Preparation of structures along with mutation of the residues were carried out using Swiss-Pdb Viewer (DeepView) version 4.01.^78^ Only one of the complexes in the reported crystal structure was used (chains B and D) for further analysis. The residues at the RON-MSPβ interface were inferred based on crystal structure reported by Chao and collaborators^76^ in both ligand and receptor were virtually mutated to alanine as listed in Table 1.

**Table 1:** List of residues mutated to alanine on RON and MSP

**RON**	**MSP**
Glu^190^	Arg^521^
Gln^193^	Cys^527^
Ser^195^	His^528^
Arg^220^	Ser^565^
Glu^287^	Arg^639^
Pro^288^	Glu^644^
Glu^289^	Glu^658^
His^424^	Arg^683^
Glu^190^/ Ser^195*^	Arg^639^/ Glu^644*^

(* Double mutation)

### Ligand-receptor binding free energy calculations using MM-GBSA method

Energy minimization and binding free energy calculation were performed using the Assisted Model Building with Energy Refinement (AMBER) suite of programs (version 14)^79^ operating on a Linux-based (Centus 6.8) GPU work station consisting of four Nvidia K40 M (each has 12 GB RAM and 2880 cuda cores), 2X Intel Xeon E5-2697 v2, 2.7 GHz (total of 48 cores), total RAM = 128 GB.

The energy minimization of RON-MSPb complex was carried out using AMBER-ff99 force field.^80^ Briefly, the usable coordinate files for AMBER (i.e. *.prmtop and *.inpcrd) were generated using leap module. Then, a correct number of counter ions (Na^+^ or Cl^-^) was added for neutralizing the total charge of the system followed by solvation of the system using TIP3P water molecules in a rectangular box with the buffering distances set to 12 Å in all directions. After that, the solvated system was submitted to an initial energy minimization process by applying Sander module (500 steps of steepest descent followed by 500 steps of conjugate gradient) followed by a 50 ps heating step where the temperature was gradually increased from 0 to 300 °K. After 50 ps of density equilibration, 500 ps of constant pressure equilibration at 300 °K with a time step of 2 fs was performed. Only bond lengths involving hydrogen atoms were constrained using the SHAKE algorithm. Final molecular dynamic simulation was individually performed for a range of 1 to 10 ns by applying the Particle Mesh Ewald (PME) method to calculate long-range electrostatic interactions. All calculations were done under periodic boundary condition where no constraint was applied to either the protein or the ligand molecules. The trajectory of the dynamic simulation was achieved by writing out the coordinates every 10 ps. After molecular dynamic simulation on receptor-ligand complex, snapshots were taken from the molecular dynamic trajectory with an interval of 10 ps. The dielectric constant values were set to 1.0 and 80 for the interior of solute and the surrounding solvent, respectively. Binding free energy was calculated for ligand–receptor complex using MM-GBSA.^80^ The interaction energies for the snapshots were calculated while excluding water molecules and counter ions and presented as the average value in the RON-MSPb system.

## Results and Discussion

Binding free energies for the complexes of RON tyrosine kinase receptor and its ligand (i.e. MSP) as well as the mutants of either receptor or ligand (Table 1) were calculated by applying MM-GBSA method on molecular dynamic simulation data collected at different time. For this purpose, firstly the binding free energy was calculated for the RON-MSPb wild type complex, then the residues involved in RON and MSPb interaction were mutated to alanine followed by molecular dynamic simulation and re-calculation of the binding free energy for different time intervals ranging from 1 to 10 ns to estimate the contribution and the effect of individual residues in RON and MSPb binding. The binding free energies (ΔG_bind_) for wild type and mutant forms were calculated as follows:

ΔG_bind_ = G_water_ (complex) - G_water_ (receptor) - G_water_ (ligand)

where G_water_ (complex), G_water_ (receptor), and G_water_ (ligand) denote the free energies of the complex, receptor, and ligand, respectively. The free energy (ΔG) for each term is calculated using following equation:

G_molecule_ = E_gas_ + ΔG_solvation_‏- TS

ΔG_solvation_ = ΔG_GB_ + ΔG_non-polar_

E_gas_ = E_int_+ E_vdw_ + E_elec_

E_int_ = E_bond_+ E_angle_+ E_tors_

where G is the calculated average free energy, E_gas_ is the standard force-field energy, including internal energy (E_int_) in the gas phase as well as non-covalent van der Waals (E_vdw_) and electrostatic (E_elec_) energies. E_bond_, E_angle_, and E_tors_ demonstrate the contributions to the internal energy caused by the strain from the deviation of the bonds, angle, and torsion angle from their equilibrium values. ΔG_solvation_ is the solvation-free energy calculated with a numerical solution of the Poisson–Boltzmann equation and an estimate of the non-polar free energy using a surface area term.^81,82^

**Figure 1:**
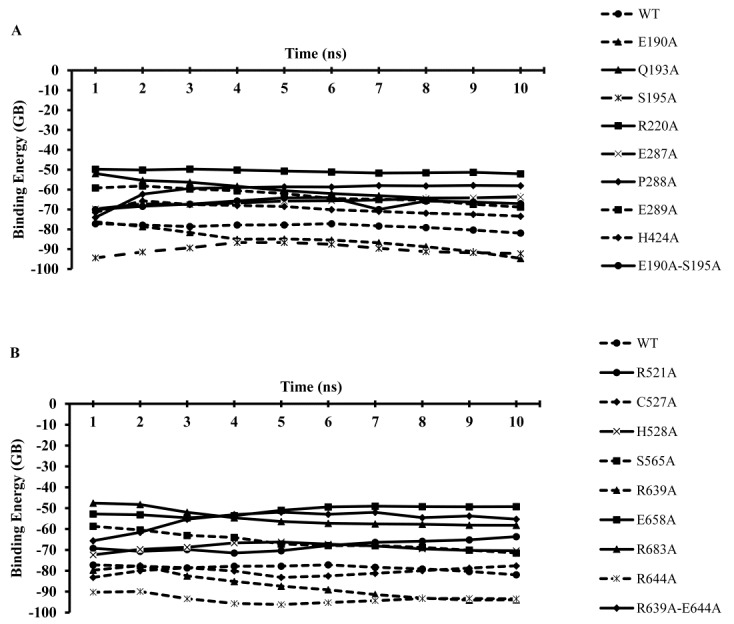
The plot of binding free energies (ΔG) for the complexes of RON-MSPb during different MD simulation time lengths (1–10 ns) using MM-GBSA calculation methods implemented in AMBER.

Figure 1 shows the results of binding free energy calculations for the complex of wild type RON and MSPb and their mutant forms using MM-GBSA method applied to molecular dynamic simulations ranging from 1 to 10 ns. These results have been also illustrated in Table 2. Results for ΔΔG binding (ΔG_Binding-wild type_ - ΔG_Binding-mutant_) for RON and MSPb are also available in Table 3. The details of all calculations for mutants and wild types of receptor and ligand are available in appendices 1 and 2.

Cancer is one of the most important causes of death in the world^83^ and several strategies including pharmacotherapy protocols are employed to control this devastating condition.^84^ Due to the importance of protein-protein interactions in cancer initiation and development, many efforts have been dedicated to target cancer cells by inhibition of those PPIs involved in cancer progression.^5,85,86^ RON a tyrosine kinase receptor has gained considerable attention as promising target in cancer therapy.^68^ Most of the therapeutic agents developed so far against RON interfere with RON and MSP binding highlighting the importance of PPIs.^69^ Therefore, the identification of hot spots involved in the interface of RON-MSP complex is of great importance in rational drug design.

**Figure 2:**
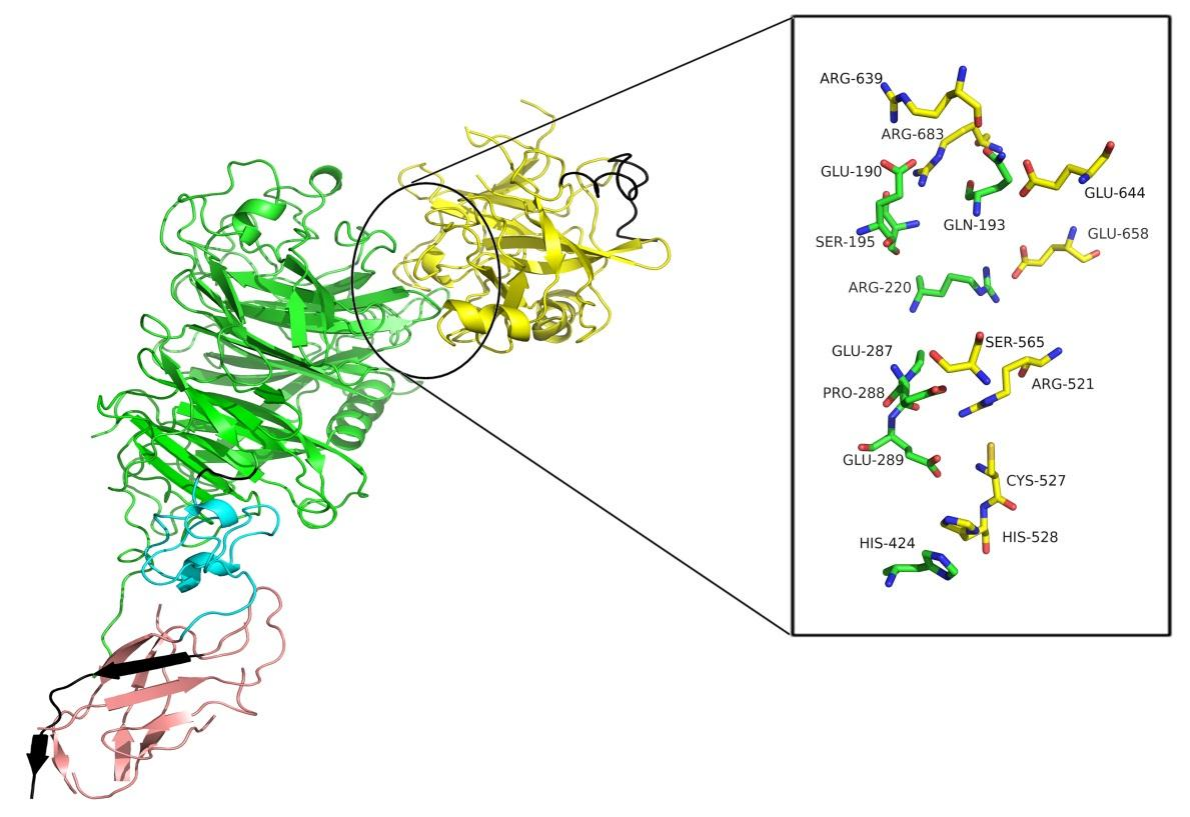
Cartoon and stick representation of RON-MSPb complex generated in PyMol (version 1.5.0.3).

In the current study, the residues reported to be involved in RON-MSPb interactions (Figure 2) were virtually mutated to alanine one at the time to determine the contribution of each residue using MM-GBSA approach. The binding free energy difference between the mutant and the wild type complexes was obtained as follows:

ΔΔG_Binding_ = ΔG_Binding-wild type_ - ΔG_Binding-mutant_

In this expression, negative ΔΔG_binding_ value implies that the substitution of the corresponding amino acid with alanine is an unfavorable substitution whereas a positive value indicates a favorable substitution in terms of binding free energy compared to the wild type complex.^87^

**Table 2:** Effects of alanine substitution on RON (A) and MSP (B) to contribution of binding energy (∆G_Bind_) for RON-MSP complex calculated using MM-GBSA method in a 1 to 10 ns molecular dynamic simulation.

		1ns	2ns	3ns	4ns	5ns	6ns	7ns	8ns	9ns	10ns	1ns
A)	**WT**	-77.17	-77.85	-78.58	-77.81	-77.78	-77.17	-78.33	-79.10	-80.36	-81.91
	**E** ^190^ **A**	-76.19	-78.62	-81.58	-85.02	-84.85	-85.41	-86.74	-88.76	-91.26	-94.65
	**Q** ^193^ **A**	-51.91	-55.39	-56.27	-58.29	-60.57	-62.00	-63.08	-64.22	-64.15	-63.56
	**S** ^195^ **A**	-94.36	-91.40	-89.31	-86.62	-86.64	-87.50	-89.57	-91.20	-91.76	-92.19
	**R** ^220^ **A**	-49.73	-50.15	-49.67	-50.17	-50.67	-51.18	-51.70	-51.52	-51.33	-52.06
	**E** ^287^ **A**	-69.70	-67.19	-67.55	-66.75	-65.81	-65.60	-65.24	-64.51	-64.20	-63.79
	**P** ^288^ **A**	-74.01	-62.30	-59.40	-59.00	-58.62	-58.67	-58.01	-58.14	-57.92	-58.12
	**E** ^289^ **A**	-59.17	-58.18	-59.67	-60.61	-61.96	-64.52	-64.92	-65.35	-67.40	-68.80
	**H** ^424^ **A**	-71.45	-65.70	-67.52	-67.99	-68.51	-70.10	-70.94	-71.88	-72.46	-73.37
	**E** ^190^ **A/S** ^195^ **A**	-70.09	-68.40	-67.30	-65.73	-64.15	-64.02	-69.99	-65.81	-66.26	-67.10
B)	**WT**	-77.17	-77.85	-78.58	-77.81	-77.78	-77.17	-78.33	-79.10	-80.36	-81.91
	**R** ^521^ **A**	-69.23	-70.67	-69.80	-71.45	-70.41	-67.89	-66.31	-65.83	-65.14	-63.65
	**C** ^527^ **A**	-83.18	-79.98	-78.55	-80.10	-83.16	-82.49	-81.23	-79.92	-78.73	-77.63
	**H** ^528^ **A**	-72.37	-69.73	-68.66	-66.65	-66.17	-67.20	-68.09	-69.49	-70.27	-70.38
	**S** ^565^ **A**	-58.67	-60.44	-63.05	-64.01	-67.13	-67.90	-67.93	-68.80	-70.12	-71.47
	**R** ^639^ **A**	-79.74	-77.52	-82.47	-85.12	-87.36	-89.09	-91.37	-93.07	-94.06	-93.91
	**E** ^644^ **A**	-90.27	-89.90	-93.40	-95.66	-96.18	-95.22	-94.30	-93.31	-93.29	-93.42
	**E** ^658^ **A**	-52.77	-53.15	-54.63	-53.59	-51.00	-49.41	-49.05	-49.24	-49.31	-49.28
	**R** ^683^ **A**	-47.54	-48.21	-51.97	-54.61	-56.40	-57.25	-57.53	-57.68	-58.10	-58.24
	**R** ^639^ **A/E** ^644^ **A**	-65.58	-61.56	-55.29	-53.06	-51.88	-52.91	-51.99	-54.55	-53.77	-55.28

**Table 3:** The binding energy differences (DDG_binding_= DG_wildtype_ -DG_mutant_) for wild type and mutant forms of RON-MSP complex. The mutations are performed on RON (A) and MSP (B) using *in silico* alanine substitution.

		**1ns**	**2ns**	**3ns**	**4ns**	**5ns**	**6ns**	**7ns**	**8ns**	**9ns**	**10ns**
**A)**	**E** ^190^ **A**	-0.98	0.78	3.01	7.21	7.07	8.24	8.41	9.66	10.90	12.74
	**Q** ^193^ **A**	-25.26	-22.45	-22.30	-19.52	-17.22	-15.17	-15.25	-14.88	-16.21	-18.35
	**S** ^195^ **A**	17.19	13.55	10.73	8.81	8.86	10.33	11.25	12.11	11.41	10.28
	**R** ^220^ **A**	-27.44	-27.70	-28.90	-27.64	-27.12	-25.99	-26.63	-27.58	-29.03	-29.85
	**E** ^287^ **A**	-7.47	-10.65	-11.03	-11.06	-11.97	-11.57	-13.09	-14.59	-16.16	-18.12
	**P** ^288^ **A**	-3.16	-15.54	-19.18	-18.81	-19.17	-18.50	-20.32	-20.96	-22.44	-23.79
	**E** ^289^ **A**	-18.00	-19.67	-18.90	-17.19	-15.82	-12.64	-13.41	-13.75	-12.96	-13.12
	**H** ^424^ **A**	-5.72	-12.15	-11.05	-9.82	-9.27	-7.06	-7.38	-7.22	-7.90	-8.54
	**E** ^190^ **A/ S** ^195^ **A**	-7.08	-9.44	-11.28	-12.08	-13.63	-13.15	-8.33	-13.28	-14.10	-14.81
**B)**	**R** ^521^ **A**	-7.94	-7.17	-8.77	-6.36	-7.37	-9.28	-12.01	-13.26	-15.21	-18.26
	**C** ^527^ **A**	6.01	2.14	-0.03	2.29	5.37	5.32	2.90	0.82	-1.62	-4.28
	**H** ^528^ **A**	-4.80	-8.12	-9.91	-11.16	-11.61	-9.97	-10.24	-9.60	-10.09	-11.53
	**S** ^565^ **A**	-18.50	-17.40	-15.53	-13.80	-10.66	-9.27	-10.40	-10.30	-10.24	-10.44
	**R** ^639^ **A**	2.57	-0.33	3.89	7.31	9.58	11.93	13.04	13.97	13.70	12.00
	**E** ^644^ **A**	13.10	12.06	14.82	17.85	18.40	18.06	15.98	14.21	12.94	11.51
	**E** ^658^ **A**	-24.40	-24.70	-23.95	-24.21	-26.78	-27.76	-29.28	-29.86	-31.04	-32.63
	**R** ^683^ **A**	-29.63	-29.64	-26.61	-23.19	-21.38	-19.92	-20.79	-21.42	-22.26	-23.68
	**R** ^639^ **A-E** ^644^ **A**	-11.59	-16.29	-23.29	-24.75	-25.91	-24.26	-26.33	-24.55	-26.59	-26.63

The results of molecular dynamic simulation of RON indicated that all receptor (except for Glu^190^ and Ser^195^) and ligand (except for Arg^639^ and Glu^644^) mutants have low affinity compared to the wild type as deduced from the negative ΔΔG values shown in Figure 2 and Table 3.

One of the crucial residues at the interface of RON-MSPb complex is RON Gln^193^; its side chain NH_2_ group makes two ionic interactions with carboxylate group of MSP Glu^644^and carbonyl group of Arg^639^. In addition, Arg^639^of MSPis involved in another interaction with RON Glu^1^ which will be discussed later.^76^ The MM-GBSA based binding energy calculations on the wild type and Q^193^A mutant showed that this amino acid is important in the binding (also confirmed by Chao and coworkers)^76^ while the calculations did not support the importance of its partners MSP, i.e. Arg^639^ and Glu^644^. To shade more light on this issue, an *in silico* R^639^A/E^644^A double mutation was introduced on MSP and then the binding energy calculated. Surprisingly, results showed that the double mutation caused unfavorable effect on binding energy for RON-MSPβ complex formation highlighting the importance of simultaneous interaction established between both Arg^639^ and Glu^644^with Gln^193^.

The RON Arg^220^ is another key residue involved in charge-charge interaction with Glu^658^of MSP.^76^ The ΔΔG values calculated for R^220^A mutant during 1 to 10 ns molecular dynamic simulation range from ~ -25 to -30 Kcal/mol, which are the highest negative values obtained for all RON mutants. This observation implies the great importance of this residue as a hot spot in the interaction between RON and MSPb. Interestingly, the ΔΔG values for E^658^A mutant has also high negative value (Table 2 and 3). This is in agreement with experimental observation reported previously.^76^

According to the study of Chao et al, MSP Arg^521^ simultaneously interacts with three residues of RON namely Glu^287^, Pro^288^and Glu^289^.^76^ Additionally, RON Glu^287^ forms a hydrogen bond interaction with the hydroxyl group of MSP Ser^656^ whereas Glu^289^ of RON establishes an ionic interaction with MSP His^528^ as well as interaction with the backbone NH group of MSP Cys^527^. Moreover, MSP His^528^, located in proximity of RON Glu^289^ is engaged in aromatic interaction with His^424^. The results of computational alanine scanning reported here revealed that Glu^287^, Pro^288^, Glu^289^, and His^424^ of RON located at the interface of RON-MSPb complex are crucial residues for its binding to MSPb.^76^ According to ΔΔG values, Pro^288^, Glu^287^, and Glu^289^ of RON are the next most important amino acids after Arg^220^ (see Table 3).It seems that the importance of these residues is related to their interactions with more than one residues on MSP (except for Pro^288^). In the case of Pro^288^, it interacts only with MSP Arg^521^ which in turn is highly important due to its participation in multiple interactions with RON Glu^287^, and Glu^289^.^76^ Based on binding ΔΔG values, His^424^ seems to be less important in comparison to other RON residues at the interface. However, this residue can also be considered as a hot spot on RON (Table 2 and 3). Additionally, MSP His^528^ and Ser^565^ are suggested to be important residues for RON binding despite the fact that their ΔΔG values are not as significant as those mentioned above (Table 2 and 3). The ΔΔG binding calculated for MSP Cys^527^ using different molecular dynamic simulation intervals includes both positive (1 to 8 ns) and negative (9 to 10 ns) values, making it difficult to extrapolate its importance in the binding. It seems that interaction via Cys^527^switches on and off during molecular dynamic simulation. However, the ΔΔG values toward end of simulation reach -4 kcal/mol which indicates positive contribution of this residue in RON-MSP binding.

E^190^A and S^195^A mutations can be considered exception as the binding affinities toward ligand were improved after mutation to alanine. The crystallography studies on RON-MSPb complex showed that RON Glu^190^ is involved in two salt bridges via its carboxylate group with guanidinium group of Arg^639^ and Arg^683^,^76^ however, our results do not attribute positive contribution for this residue as inferred from its positive ΔΔG values in MM-GBSA calculations upon mutation to alanine (See Table 3). Such disagreement between the reported experimental results and our *in silico* estimates may be due to the fact that Glu^190^ interacts with two different MSP residues (i.e. Arg^639^ and Arg^683^), which are already interacting with other RON residues.^76^ Therefore, lack of their interactions with Glu^190^may not contribute favorably in the overall binding energy.

RON Ser^195^ is shown to be involved in a charge-charge interaction with MSP Arg^683^,^76^ however, our results did not identify this amino acid as an important residue in RON-MSPb complex (Table 3). Again the disagreement between our *in silico* estimates and the crystallographic data may be due to the formation of another interaction by Arg^683^ with RON via Glu^190^ which renders the interaction between Ser^195^ and Arg^683^ less important.^76^ The only previous experimental site directed mutagenesis studies on the residues at the interface of RON-MSP complex was carried out for Arg^683^ and results obtained are in agreement with the ones discussed below.^75^ This amino acid is an important residue in the interaction of RON-MSPβ complex based on *in silico* calculation despite the results obtained for its partners on MSP (i.e Glu^190^ and Ser^195^). In order to gain more information regarding these residues an *in silico* double mutation (E^190^A/S^195^A) study was performed. This double mutation lead to a positive ΔΔG value indicative of their harmonic interplay in the interaction with Arg^683^.

## Conclusion

In modern drug design and discovery process, computational approaches have streamlined a promising perspective by supplying useful and supportive information. In this context, identification of hot spots in biomolecules’ interactions through the estimating the binding affinity of molecules towards targets of interest can provide valuable information where protein-protein interactions are important initiators in cancer pathogenesis. Virtual alanine scanning mutagenesis is one of the tools that are commonly employed for this purpose. Therefore, in the current investigation, amino acids reported to be at the interface of RON-MSPb complex were evaluated using the MM-GBSA method and some of them were assigned as hot spots in the interaction. Taken together, in *silico* alanine scanning mutagenesis results revealed that Gln^193^, Arg^220^, Glu^287^, Pro^288^, Glu^289^ and His^424^ residues from RON and Arg^521^, His^528^, Ser^565^, Glu^658^, and Arg^683^ form MSPβ may play important roles in protein-protein interaction between RON and MSP. Identification of these RON hot spots is important in designing anti-RON drugs when the aim is disruption of RON-MSP interaction. In the same way, the acquired information regarding the critical amino acids of MSPβ can be used in the process of rational drug design for developing MSP antagonizing agents, the development of novel MSP mimicking peptides where inhibition of RON activation is required, and the design of experimental site directed mutagenesis studies.

## Acknowledgments

This work is a part of Ph.D thesis of Omid Zarei at Tabriz university of Medical Sciences.

## Ethical Issues

Not applicable.

## Conflict of Interest

The authors declare no conflict of interests.
